# Characterization of exposure–response relationships of ipatasertib in patients with metastatic castration-resistant prostate cancer in the IPATential150 study

**DOI:** 10.1007/s00280-022-04488-2

**Published:** 2022-10-28

**Authors:** Naoki Kotani, Justin J. Wilkins, Janet R. Wade, Steve Dang, Dhruvitkumar S. Sutaria, Kenta Yoshida, Sameer Sundrani, Hao Ding, Josep Garcia, Heather Hinton, Rucha Sane, Pascal Chanu

**Affiliations:** 1 Genentech, Inc., South San Francisco, CA USA; 2 Pharmaceutical Science Department, Chugai Pharmaceutical Co., Ltd., 1-1 Nihonbashi-Muromachi 2-Chome, Chuo-ku, Tokyo, 103-8324 Japan; 3 Occams Coöperatie UA, Amstelveen, The Netherlands; 4 Department of Bioengineering/Biomedical Computation, Stanford University, Stanford, CA USA; 5 F. Hoffmann-La Roche AG, Basel, Switzerland; 6 Department of Clinical Pharmacology, Genentech/Roche, Lyon, France

**Keywords:** Ipatasertib, AKT inhibitor, Metastatic castration-resistant prostate cancer, IPATential150, Exposure–response

## Abstract

**Purpose:**

The exposure–response relationships for efficacy and safety of ipatasertib, a selective AKT kinase inhibitor, were characterized using data collected from 1101 patients with metastatic castration-resistant prostate cancer in the IPATential150 study (NCT03072238).

**Methods:**

External validation of a previously developed population pharmacokinetic model was performed using the observed pharmacokinetic data from the IPATential150 study. Exposure metrics of ipatasertib for subjects who received ipatasertib 400 mg once-daily orally in this study were generated as model-predicted area under the concentration–time curve at steady state (AUC_SS_). The exposure–response relationship with radiographic progression-free survival (rPFS) was evaluated using Cox regression and relationships with safety endpoints were assessed using logistic regression.

**Results:**

A statistically significant correlation between ipatasertib AUC_SS_ and improved survival was found in patients with PTEN-loss tumors (hazard ratio [HR]: 0.92 per 1000 ng h/mL AUC_SS_, 95% confidence interval [CI] 0.87–0.98, *p* = 0.011). In contrast, an improvement in rPFS was seen in subjects receiving ipatasertib treatment (HR: 0.84, 95% CI 0.71–0.99, *p* = 0.038) but this effect was not associated with ipatasertib AUC_SS_ in the intention-to-treat population. Incidences of some adverse events (AEs) had statistically significant association with ipatasertib AUC_SS_ (serious AEs, AEs leading to discontinuation, and Grade ≥ 2 hyperglycemia), while others were associated with only ipatasertib treatment (AEs leading to dose reduction, Grade ≥ 3 diarrhea, and Grade ≥ 2 rash).

**Conclusions:**

The exposure–efficacy results indicated that patients receiving ipatasertib may continue benefiting from this treatment at the administered dose, despite some variability in exposures, while the exposure–safety results suggested increased risks of AEs with ipatasertib treatment and/or increased ipatasertib exposures.

**Supplementary Information:**

The online version contains supplementary material available at 10.1007/s00280-022-04488-2.

## Introduction

Ipatasertib is a potent, selective, adenosine triphosphate-competitive, small-molecule inhibitor of the activated form of Akt that disrupts oncogenic phosphoinositide 3-kinase (PI3K)/Akt signaling [1, 2]. It has been developed as a single agent and in combination with other therapies for the treatment of several types of cancers including metastatic castration-resistant prostate cancer (mCRPC). Within the context of prostate cancer, the PI3K–Akt–mammalian target of rapamycin (mTOR) pathway is one of the most frequently activated pathways, with genomic alterations occurring in approximately 50% of patients with prostate cancer [3]. The majority of pathway alterations are due to phosphatase and tensin homolog (PTEN) loss or mutations, and studies have consistently demonstrated that low PTEN expression or PTEN loss may be associated with worse prognosis [4–12], regardless of whether patients are newly diagnosed, receiving treatment for localized disease, or have mCRPC.

The Phase 2 study (A.MARTIN, NCT01485861) which investigated the combination of ipatasertib (400 or 200 mg) with the CYP17 inhibitor abiraterone (1000 mg) once-daily (QD) orally in patients with mCRPC in the second or later line settings showed prolongation of radiographic progression-free survival (rPFS) in the ipatasertib cohort vs placebo, with a larger rPFS prolongation observed in PTEN-loss tumors vs those without [13]. The exposure–response relationships of ipatasertib in the A.MARTIN study population were characterized and quantitative benefit–risk assessment using a clinical utility index approach was conducted to support ipatasertib Phase 3 dose selection in patients with mCRPC; as a consequence, ipatasertib 400 mg QD, showing the highest probability of achieving better benefit–risk balance than other doses, was selected for further development [14].

More recently, the efficacy and safety of ipatasertib in patients with mCRPC in the first-line setting were investigated in a large, multicenter, randomized, double-blind, Phase 3 trial (IPATential150 study, NCT03072238) [15]. Patients were randomly assigned 1:1 to receive ipatasertib (400 mg QD) plus abiraterone (1000 mg QD) and prednisolone (5 mg twice a day [BID]) or placebo plus abiraterone and prednisolone (with the same dosing schedule). A total of 1101 patients were enrolled to the study (554 were assigned to the placebo arm and 547 to the ipatasertib arm), and in the 521 patients (261 in the placebo arm and 260 in the ipatasertib arm) who had tumors with PTEN-loss, median rPFS was 16.5 months (95% confidence interval [CI] 13.9–17.0) in the placebo arm and 18.5 months (95% CI 16.3–22.1) in the ipatasertib arm (hazard ratio [HR]: 0.77 [95% CI 0.61–0.98]; *p* = 0.034) which suggested that combined AKT and androgen–receptor signaling pathway inhibition with ipatasertib and abiraterone is a potential treatment for men with PTEN-loss mCRPC. The efficacy trend was also observed in the intention-to-treat (ITT) population, while it did not reach prespecified significance at α = 0.01 (median rPFS was 16.6 months [95% CI 15.6–19.1] in the placebo arm and 19.2 months [95% CI 16.5–22.3] in the ipatasertib arm; HR: 0.84 [95% CI 0.71–0.99]; *p* = 0.043).

In this study, the pharmacokinetic (PK) profiles of ipatasertib and its major metabolite M1 (also known as G-037720) in patients with mCRPC in the IPATential150 study population were investigated and exposure metrics in these patients were derived using a previously developed population PK model [16]; subsequently, we aimed to characterize exposure–efficacy and –safety relationships of ipatasertib 400 mg QD in the IPATential150 study population.

## Materials and methods

### Data and study design

The IPATential150 study was a Phase 3, randomized, double-blind, placebo-controlled, multicenter trial testing ipatasertib plus abiraterone plus prednisone/prednisolone, relative to placebo plus abiraterone plus prednisone/prednisolone in adult male patients with asymptomatic or mildly symptomatic, previously untreated mCRPC [15]. The study was approved by an ethics committee or institutional review board at each trial site and carried out in accordance with the International Conference on Harmonization Guideline for Good Clinical Practice. Written informed consent was obtained from all subjects before enrollment in the trial.

Patients who met the eligibility criteria were randomized in a 1:1 ratio to one of the two treatment arms; patients in the experimental arm received ipatasertib (400 mg QD) and patients in the control arm received matching placebo, each consisting of 28-day cycles of oral administration. The same formulation of ipatasertib (a film-coated tablet), which was also used in the randomized Phase 2 part of a Phase 1b/2 A.MARTIN study [13], was used throughout the study. In addition, all patients received abiraterone (1000 mg QD) plus prednisone/prednisolone (5 mg BID). Treatments were continued until disease progression (as assessed by Response Evaluation Criteria in Solid Tumors [RECIST] version 1.1 [17], or Prostate Cancer Working Group 3 [PCWG3] criteria [18], or both), intolerable toxicity, elective withdrawal or study conclusion. The co-primary endpoints were investigator-assessed rPFS in patients with PTEN-loss tumors and in the ITT population. Safety was evaluated in all patients who received any dose of ipatasertib, abiraterone, or placebo according to the Common Terminology Criteria for Adverse Events, version 4.0. Blood samples for PK assessments of ipatasertib and its major metabolite M1 were collected on days 1 (1–3 h post-dose) and 15 (pre-dose and 1–3 h post-dose) of cycle 1, day 1 of cycle 3 (pre-dose and 1–3 h post-dose), and day 1 of cycle 6 (pre-dose).

### External validation of IPATential150 PK data and generation of exposure metrics

A previously developed population PK models of ipatasertib and M1 [16] were applied to the observed individual PK data from the IPATential150 study, which were not part of the data used for model development (i.e., served as external validation of the models), and empirical Bayes estimates (EBEs) of model parameters for each patient in the ipatasertib arm of the study were generated. Data from a total of 546 individuals who received at least one dose of ipatasertib and had at least one quantifiable PK observation were included in the analysis, comprising a total of 2561 ipatasertib observations and 2542 M1 observations. These EBEs were in turn used to simulate a concentration–time profile for the intervals required, from which the necessary exposure metrics were derived. A dosing interval and observation period of 24 h were assumed; concentration–time profile at 0–24 h after the initial dose and at 336–360 h after 2 weeks of QD dosing was used to derive exposure metrics after the first dose (i.e., single dose) and at steady state, respectively. The planned (per protocol) nominal dosing of 400 mg QD was always assumed and any dose adaptations were not taken into account to avoid potential post-randomization bias in the analysis (e.g., correlations between dose alterations and response). Generated exposure metrics included area under the concentration–time curve (AUC, calculated by the linear trapezoidal rule), maximum concentration (*C*_max_), assigned as the peak concentration within the specified interval, and trough concentration (*C*_min_), assigned as the concentration at 24 h (for the single dose) and at 360 h (for the steady state), after the single dose (AUC_SD_, *C*_max,SD_, *C*_min,SD_) and at steady state (AUC_SS_, *C*_max,SS_, *C*_min,SS_). Correlations between the simulated PK parameters (AUC_SD_, *C*_max,SD_, *C*_min,SD_, AUC_SS_, *C*_max,SS_, and *C*_min,SS_) as well as each patient’s AUC_SS_ values for ipatasertib and M1 were evaluated; if these were highly correlated, only the representative exposure metrics (e.g., ipatasertib AUC_SS_) were to be used in subsequent analysis. Zero exposures were assigned to 554 patients in the control arm of the IPATential150 study.

### Exposure–efficacy analysis on rPFS

The endpoint of interest for exposure–efficacy analysis was rPFS, assessed both in patients with PTEN-loss tumors and in the ITT population. Radiographic PFS was defined as the time from the date of randomization to the first occurrence of documented disease progression, as assessed by the investigators with the use of the PCWG3 criteria [18] (soft tissue by computerized tomography or magnetic resonance imaging scans according to the RECIST v1.1 [17], and bone metastasis by bone scan according to the PCWG3 criteria) or death from any cause, whichever occurs first. As an exploratory graphical analysis, rPFS data were explored using Kaplan–Meier plots with stratification by ipatasertib exposure groups; subjects in the placebo arm were compared with subjects from the active treatment arms who were grouped in exposure quartiles. Modeling of rPFS was performed using the Cox proportional hazards model, which allows the fitting of univariable and multivariable regression models with survival outcomes, as follows:

1 $$ h\left( {t\left| {X_{i} } \right.} \right) = h_{0} \left( t \right) \times \exp \left( {\beta_{1} \times X_{i,1} + \cdots + \beta_{n} \times X_{i,n} } \right). $$

Here, *h*(*t*) is the hazard (the instantaneous rate at which events occur), *h*_0_(*t*) is the underlying baseline hazard, *X*_*i*_ is the set of explanatory covariates for individual *i*, *β*_1*…n*_ are the coefficients describing the effects of explanatory covariates 1 − *n*, and *X*_*i,*1*…i,n*_ are explanatory covariates values 1 − *n* in individual *i*.

Covariate testing was performed in a stepwise fashion. Each putative covariate relationship was fitted in a univariable model first. All those with a *p* value for inclusion of < 0.15 were jointly included in a “full” model. Each was then subsequently removed one at a time and assessed using the Akaike information criterion (AIC) estimated as follows:

2 $$\mathrm{AIC}=2k-2ln(\widehat{L}).$$

Here, *k* is the number of estimated parameters and *L̂* is the value of the likelihood function. When no further relationships met the retention criterion (i.e., removing a term in the model reduced the AIC relative to the model including the term), the model was considered final.

### Time-varying covariate Cox modeling analysis for rPFS

In addition to the exposure–efficacy analysis using the standard Cox proportional hazards model, time-varying covariate Cox modeling analysis for rPFS was performed for ipatasertib-treated patients in the ITT population. The analysis was performed with Cox regression approach, but using the daily ipatasertib dose at each timepoint as a covariate instead of using one fixed value (i.e., nominal dose) per patient to calculate hazard. For the patients who discontinued ipatasertib due to adverse events (AEs), daily dose of 0 mg was assigned after the dose discontinuation. For the patients who discontinued ipatasertib due to other reasons (but before record of PFS event), PFS status was censored at the treatment discontinuation date.

### Exposure–safety analysis on safety outcomes

Exposure–safety relationships were assessed for the following endpoints; serious AEs (SAEs), AEs leading to treatment discontinuation, AEs leading to dose reduction, and specific AEs of clinical interest (which were the following identified risks of ipatasertib) including diarrhea (Grade ≥ 2 and Grade ≥ 3), hyperglycemia (Grade ≥ 2), and rash (Grade ≥ 2). All safety events were binary outcomes (event or no event) and analysis was conducted using logistic regression as follows:

3 $$ Z_{i} = {\text{ln}}\left( {\frac{{p\left( {y = 1} \right)}}{{1 - p\left( {y = 1} \right)}}} \right) = \beta_{0} + \beta_{1} \times X_{i,1} + \cdots + \beta_{n} \times X_{i,n} . $$

Here, *Z*_*i*_ is the log odds of the probability p that outcome *y* = 1 in individual *i*, *β*_0_ is the baseline log-odds that event *y* = 1, *β*_1*…n*_ are the coefficients describing the effects of explanatory covariates 1 − *n*, and *X*_*i,*1*…i,n*_ are explanatory covariates values 1 − *n* in individual *i*.

The odds of outcome *y* = 1 may be recovered by exponentiating the log-odds. The probability *P*_*i*_ of the event in individual *i* can be calculated as follows:

4 $${P}_{i}=\frac{1}{1+{\mathrm{e}}^{-{Z}_{i}}}$$

Every putative covariate relationship was added in a single step. Each was subsequently removed one at a time and assessed in terms of the associated AIC. When no further relationships met the retention criterion (i.e., removal of the predictor reduced the AIC relative to the model including it), the model was considered final.

### Covariate scope

Covariates included in the analyses for efficacy and safety, apart from ipatasertib treatment and exposure (e.g., AUC), included baseline age, baseline weight, race, geographic region, the Eastern Cooperative Oncology Group (ECOG) status at baseline, prior taxane-based therapy in hormone-sensitive prostate cancer setting (yes or no), factor(s) of progressive disease before initiation of the study treatment (PSA only or other), presence of visceral metastasis (yes or no), tumor PTEN-diagnostic status by immunohistochemistry assay (PTEN-loss: yes or no), baseline glucose (for hyperglycemia only), baseline HbA1c (for hyperglycemia only), and abiraterone trough concentration at steady state (pre-dose at day 15 of cycle 1; for efficacy analysis only).

Forest plots were used to illustrate the effects of covariates on parameters and generated for the model including all potential covariates of interest and for the final reduced model after stepwise reduction. For each covariate effect coefficient, the asymptotic standard errors were used to generate a 95% CI for the coefficient (defined as ± 1.96 × standard error [SE]). The point estimate and the upper and lower limits of the 95% CI for the covariate coefficient were used together with the covariate and the estimated value of the parameter to define a 95% confidence range for the parameter given the covariate relationship, expressed relative to the typical value of the population parameter in the population (such that “no effect” would be 1).

### Software

Generation of EBEs from the population PK models was performed in the nonlinear mixed effect modeling software NONMEM version 7.4.3 (ICON Development Solutions, Ellicott City, MD, USA) [19], supplemented with Perl-speaks-NONMEM (PsN) version 4.9.0 (Uppsala University, Uppsala, Sweden) [20, 21]. R software version 4.0.0 (The R Foundation, Vienna, Austria) was used to derive exposure metrics based on EBEs generated by the population PK models (for ipatasertib and M1), and for general scripting, data management, Cox proportional hazards modeling, logistic regression modeling, goodness of fit analyses, and model evaluation.

## Results

### Ipatasertib exposures in the IPATential150 study

**Fig. 1:**
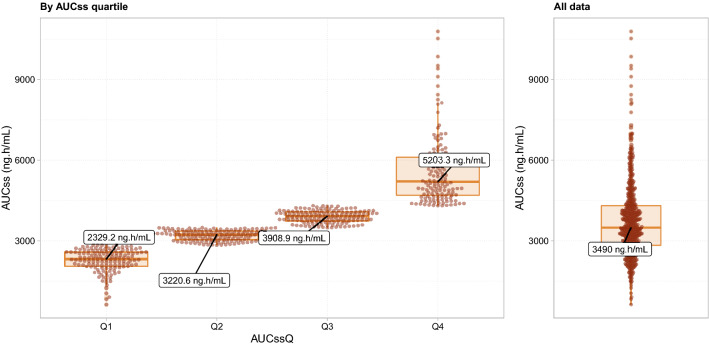
Distributions of the simulated ipatasertib AUC_SS_ in the IPATential150 study population by AUC_SS_ quartile (left panel) or as overall (right panel). Filled circles represent the simulated exposure of individual patients. Annotations represent medians in each group. Solid bold lines, shaded boxes, and whiskers in the box plots represent the medians, interquartile ranges, and 1.5 times the interquartile range, respectively. *AUC*_*SS*_ area under the concentration–time curve at steady state, *AUC*_*SS*_*Q* AUC_SS_ Quartiles

The standard goodness-of-fit plots for the ipatasertib PK data from the IPATential150 study as fitted by the previously developed population PK model using its original parameters are shown in Fig. S1. The model fitted the data adequately; the individual predictions plotted against observations suggested that exposures generated for the IPATential150 study subjects by the model would be appropriate. One very large outlier was noted, most likely the result of an error in this individual’s dosing history; however, the patient was kept in the exposure–response analysis data set anyway, since there was no clear reason to exclude the data from the analysis. A visual predictive check plot describing the ability of the previously developed population PK model of ipatasertib to reproduce the IPATential150 study data confirms the model adequacy (Fig. S2). The data points at nominal time of 2 h after the first dose, which is close to *C*_max_, were slightly underpredicted, but the ipatasertib concentrations at all other observation times were adequately reproduced by the model. The 90% ranges of observations were comparable with the model-predicted 90% range at all nominal timepoints. Distributions of the simulated ipatasertib AUC_SS_ in the IPATential150 study population are shown in Fig. 1. Exposures of ipatasertib were similar between patients with PTEN loss and non-loss (data not shown). Given that all the simulated PK parameters (AUC_SD_, *C*_max,SD_, *C*_min,SD_, AUC_SS_, *C*_max,SS_, and *C*_min,SS_) were highly correlated with each other (Fig. S3), AUC_SS_ was used as the primary exposure metric in the subsequent exposure–response analyses. Moreover, the correlation between each patient’s AUC_SS_ values for ipatasertib and M1 was high enough (*r*^2^ = 0.823, Fig. S4) to support the use of ipatasertib exposure metrics only in the subsequent exposure–response analyses.

### Exposure–efficacy of ipatasertib

**Fig. 2:**
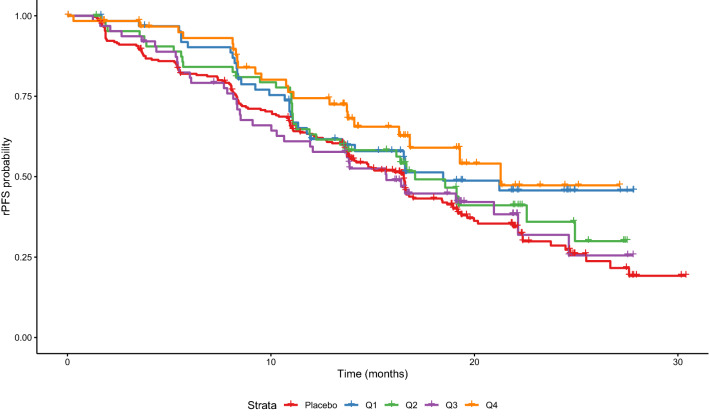
Kaplan–Meier plot for rPFS by ipatasertib AUC_SS_ quartile in patients with PTEN-loss tumors only in the IPATential150 study. The ipatasertib AUC_SS_ ranges in each quartile group were 631–2850 ng h/mL for Q1, 2850–3470 ng h/mL for Q2, 3490–4250 ng h/mL for Q3, and 4290–10,500 ng h/mL for Q4. *AUC*_*SS*_ area under the concentration–time curve at steady state, *rPFS* radiographic progression-free survival

**Fig. 3:**
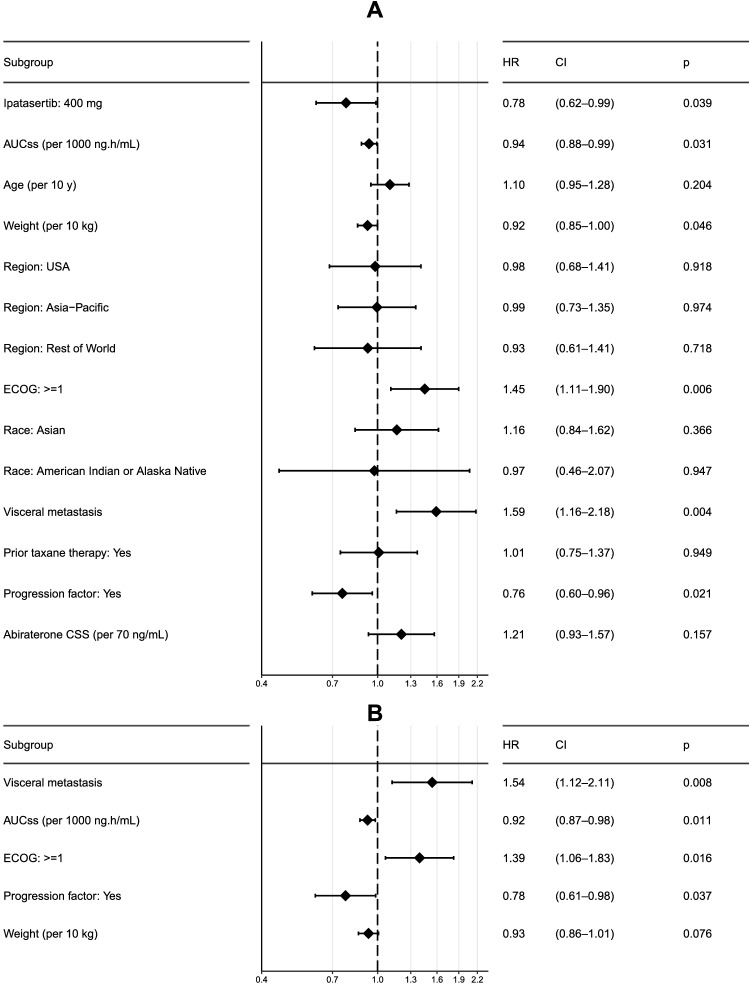
Forest plots for rPFS (point estimates and 95% CIs of HR) including all covariates of interest (**A**) and after stepwise reduction (i.e., final model; **B**) in patients with PTEN-loss tumors only in the IPATential150 study. *AUC*_*SS*_ area under the concentration–time curve at steady state, *CI* confidence interval; *C*_*SS*_ trough concentration at steady state, *HR* hazard ratio, *rPFS* radiographic progression-free survival

A Kaplan–Meier plot of rPFS in patients with PTEN-loss tumors (*n* = 521) stratified by placebo and quartiles of ipatasertib exposure is presented in Fig. 2. There appears to be a beneficial effect of ipatasertib treatment as the Kaplan–Meier curves for exposure quartile groups are generally positioned above that for placebo group; however, no clear differentiation in rPFS was seen among the exposure quartile groups. A similar trend was observed when focusing on the ITT population, which is another population of interest in the study evaluated as one of the co-primary endpoints (data not shown). Summary statistics of baseline demographics and other characteristics for the patients in the exposure–response analysis data set are provided in Table 1. A model containing all potential covariates of interest (univariate fits) was developed first as shown in Fig. 3A. The covariates with a *p* value of < 0.15 were ipatasertib treatment (HR: 0.78, 95% CI 0.62–0.99, *p* = 0.039), AUC_SS_ (HR: 0.94 per 1000 ng h/mL AUC_SS_, 95% CI 0.88–0.99, *p* = 0.031), baseline weight (HR: 0.92 per 10 kg, 95% CI 0.85–1.00, *p* = 0.046), ECOG status at baseline ≥ 1 (HR: 1.45, 95% CI 1.11–1.90, *p* = 0.006), presence of visceral metastasis (HR: 1.59, 95% CI 1.16–2.18, *p* = 0.004), and PSA only as a progression factor (HR: 0.76, 95% CI 0.60–0.96, *p* = 0.021). These covariates were jointly included in a “full” model, and the model was then subjected to stepwise reduction process, yielding the final reduced model (Fig. 3B). A statistically significant correlation between ipatasertib AUC_SS_ and improved survival was found in patients with PTEN-loss tumors (HR: 0.92 per 1000 ng h/mL AUC_SS_, 95% CI 0.87–0.98, *p* = 0.011). Other predictors of improved rPFS were PSA only as a progression factor (HR: 0.78, 95% CI 0.61–0.98) and increasing baseline weight (HR: 0.93 per 10 kg, 95% CI 0.86–1.01). ECOG status at baseline ≥ 1 (HR: 1.39, 95% CI 1.06–1.83) and presence of visceral metastasis (HR: 1.54, 95% CI 1.12–2.11) were associated with reduced rPFS.

**Table 1:** Summary of baseline demographics and other characteristics of the patients in the IPATential150 study analysis data set

Variable	Placebo	400 mg	Total
*N*	554	547	1101
Age (years)	70 (69.6) [44; 90]	69 (69.3) [47; 93]	70 (69.5) [44; 93]
Body weight (kg)	82 (84.1) [51; 166] {4}	81 (81.8) [46; 143] {1}	82 (83) [46; 166] {5}
ECOG status			
0	401 (72.4%)	421 (77.0%)	822 (74.7%)
≥ 1	150 (27.1%)	125 (22.9%)	275 (25.0%)
Race			
White	386 (69.7%)	376 (68.7%)	762 (69.2%)
Black or African American	9 (1.62%)	10 (1.83%)	19 (1.73%)
Asian	109 (19.7%)	110 (20.1%)	219 (19.9%)
American Indian or Alaska Native	16 (2.89%)	15 (2.74%)	31 (2.82%)
Native Hawaiian or Other Pacific Islander	1 (0.181%)	1 (0.183%)	2 (0.182%)
Unknown	33 (5.96%)	35 (6.4%)	68 (6.18%)
Region			
United States	74 (13.4%)	70 (12.8%)	144 (13.1%)
Asia–Pacific	141 (25.5%)	136 (24.9%)	277 (25.2%)
EU	280 (50.5%)	281 (51.4%)	561 (51%)
Rest of World	59 (10.6%)	60 (11%)	119 (10.8%)
Visceral metastasis			
No visceral metastasis	476 (85.9%)	473 (86.5%)	949 (86.2%)
Visceral metastasis	78 (14.1%)	74 (13.5%)	152 (13.8%)
Tumor PTEN status			
Loss	261 (47.1%)	260 (47.5%)	521 (47.3%)
Non-loss	293 (52.9%)	287 (52.5%)	580 (52.7%)
Progression factor			
Yes	277 (50%)	273 (49.9%)	550 (50%)
No	277 (50%)	274 (50.1%)	551 (50%)
Prior taxane therapy			
Yes	99 (17.9%)	98 (17.9%)	197 (17.9%)
No	455 (82.1%)	449 (82.1%)	904 (82.1%)
Baseline glucose (mmol/L)	5.88 (6.01) [3.1; 10.6] {84}	5.8 (5.95) [0.294; 14] {82}	5.83 (5.98) [0.294; 14] {166}
Baseline HbA1c (%)	38.8 (46.1) [5.2; 589] {10}	39 (45.1) [5.4; 604] {8}	38.8 (45.6) [5.2; 604] {18}

Continuous variables are expressed as median (geometric mean) [range] {missing}. Categorical variables are expressed as count (percentage)

An analysis of rPFS in the ITT population was also performed, and a significant correlation was found between ipatasertib treatment and improved survival (HR: 0.84, 95% CI 0.71–0.99, *p* = 0.038) but this effect was not associated with ipatasertib AUC_SS_ (data not shown). Presence of visceral metastasis, progression factor, baseline ECOG status, location in the Asia–Pacific region, baseline weight, tumors with PTEN-loss, and baseline age were also identified as prognostic predictors of the ITT population.

### Time-varying dose intensity analysis for rPFS

There were 103 patients who discontinued ipatasertib due to AEs, and daily dose of 0 mg was assigned after the discontinuation for these patients. For 94 patients who discontinued ipatasertib for other reasons (but before record of PFS event), PFS status was censored at the treatment discontinuation date. Compared with the model which contains baseline covariates as identified above, the addition of daily ipatasertib dose each day as a time-varying covariate did not improve the model performance (chi-square *p* value: 0.655).

### Exposure–safety of ipatasertib

**Fig. 4:**
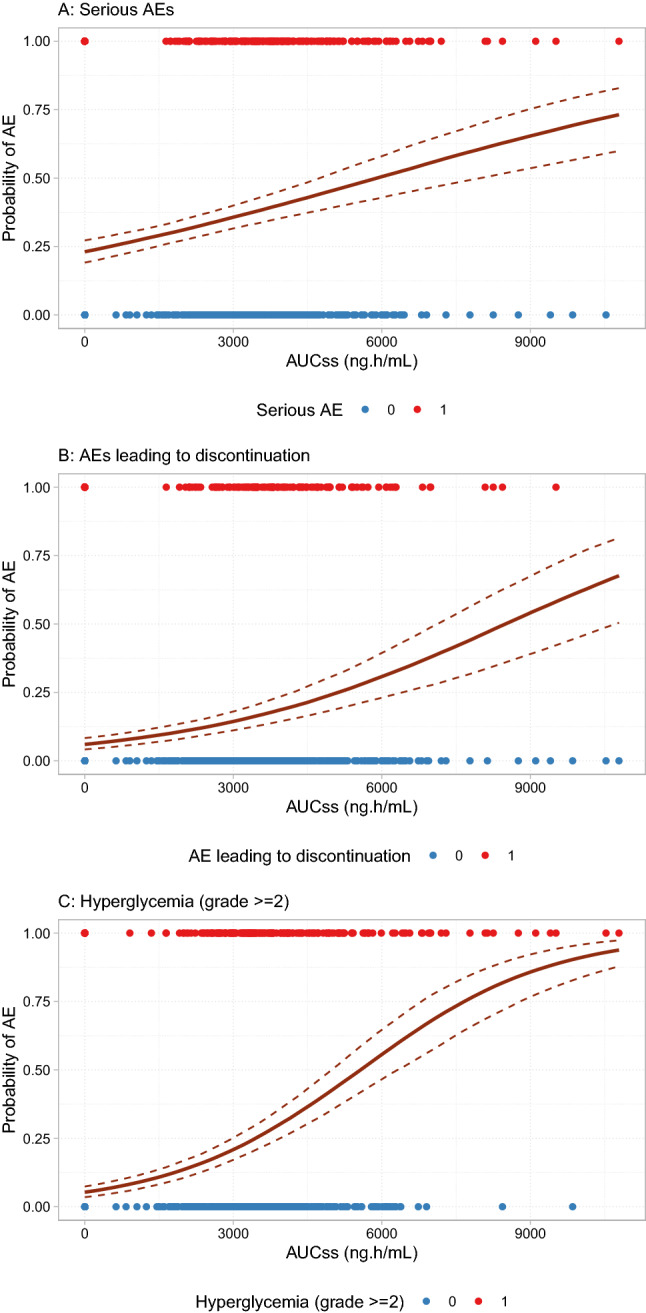
Bootstrapped model predictions (*n* = 1000) of relationship between ipatasertib exposure and safety endpoints in which ipatasertib exposure was retained in the final exposure–safety model (**A** SAE, **B** AE leading to discontinuation, **C** Grade ≥ 2 hyperglycemia). *AE* adverse event, *AUC*_*SS*_ area under the concentration–time curve at steady state, *SAE* serious adverse event

Numbers of patients (out of a total of n = 1101) reported to experience each safety event of interest in this study were as follows; 342 (31.2%) (218 [39.6%] in the active treatment arm vs 124 [22.7%] in the placebo arm) for SAEs, 144 (13.1%) (116 [21.1%] vs 28 [5.13%]) for AEs leading to treatment discontinuation, 254 (23.2%) (220 [39.9%] vs 34 [6.23%]) for AEs leading to dose reduction, 239 (21.8%) (212 [38.5%] vs 27 [4.95%]) for Grade ≥ 2 diarrhea, 61 (5.56%) (57 [10.3%] vs 4 [0.733%]) for Grade ≥ 3 diarrhea, 231 (21.1%) (190 [34.5%] vs 41 [7.51%]) for Grade ≥ 2 hyperglycemia, and 172 (15.7%) (162 [29.4%] vs 10 [1.83%]) for Grade ≥ 2 rash, respectively. Similar to the exposure-efficacy analysis, for each type of AEs, a model containing all potential covariate relationships of interest was developed first, and the model was then subjected to stepwise reduction process, yielding the final reduced model. A summary of the exposure–response analyses for safety endpoints with the final reduced models is shown in Table 2. Ipatasertib treatment, rather than exposure, was significantly associated with higher incidence of AEs leading to dose reduction (OR: 11.2, 95% CI 7.63–16.9), Grade ≥ 3 diarrhea (OR: 16.3, 95% CI 6.6–54.1), and Grade ≥ 2 rash (OR: it 25.5, 95% CI 13.6–54.6). Ipatasertib exposure, by contrast, was significantly associated with greater incidence of SAEs (OR: 1.22 per 1000 ng h/mL AUC_SS_, 95% CI 1.15–1.30), AEs leading to discontinuation (OR: 1.37 per 1000 ng h/mL AUC_SS_, 95% CI 1.26–1.49) and Grade ≥ 2 hyperglycemia (OR: 1.67 per 1000 ng h/mL AUC_SS_, 95% CI 1.52–1.84). The incidence of Grade ≥ 2 diarrhea was associated with both ipatasertib treatment (OR: 8.62, 95% CI 4.49–16.8) and exposure (OR: 1.12 per 1000 ng h/mL AUC_SS_, 95% CI 0.984–1.28). The relationship between ipatasertib exposure and SAEs, AEs leading to discontinuation, and Grade ≥ 2 hyperglycemia are illustrated as model predictions with uncertainty (i.e., bootstrapped model predictions) in Fig. 4.

**Table 2:** Summary of exposure–response modeling for safety in the IPATential150 study

Parameter	Adverse event						
	SAE	Dsc	DRd	Hgl2	Dr2	Dr3	Rsh2
Ipatasertib: 400 mg			11.2*		8.62*	16.3*	25.5*
AUCss (per 1000 ng h/mL)	1.22*	1.37*		1.67*	1.12		
Baseline age (per 10 years)	1.24*	1.25	1.41*		1.3*		
Baseline weight (per 10 kg)	0.901*	0.867*	1.13*	1.11			0.808*
Race: Asian	0.656*				0.51*	0.429	1.43
Race: American Indian/Alaska Native							0.365
Region: Asia–Pacific			1.67*				
Region: USA			1.67*				
Region: Rest of World		0.522	2.34*	1.98*	2.27*	2.45*	
Baseline ECOG: ≥ 1	1.35						
Prior taxane-based therapy		0.665				0.307*	
Visceral metastasis				0.593			0.556*
Progression factor		1.34	0.713*				
Baseline glucose (per 1 mmol/L)				1.77*			
Baseline HbA1c (per 5%)				1.63*			

Numbers are odds ratios provided by AIC reduced models. *p < 0.05

*SAE* serious adverse event, *Dsc* adverse event leading to discontinuation, *DRd* adverse event leading to dose reduction, *Hgl2* hyperglycemia (Grade ≥ 2), *Dr2* diarrhea (Grade ≥ 2), *Dr3* diarrhea (Grade ≥ 3), *Rsh2* rash (Grade ≥ 2)

Some of the covariates studied appeared to be risk factors for several of the included AEs. Increasing baseline age was associated with increasing incidence in SAEs, AEs leading to dose reduction, and Grade ≥ 2 diarrhea. Decreasing baseline weight was associated with higher incidence of SAEs, AEs leading to treatment discontinuation, and Grade ≥ 2 rash. Asian race was associated with significantly lower incidence of SAEs and Grade ≥ 2 diarrhea, and region not including the USA, the Asia–Pacific region, and the EU (“rest of world”) was associated with significantly greater incidence of AEs leading to dose reduction and Grade ≥ 2 and Grade ≥ 3 diarrhea.

## Discussion

In the present study, exposure–efficacy and –safety relationships of ipatasertib 400 mg QD in the IPATential150 study population were characterized using the observed data of the efficacy and safety endpoints as well as exposure metric in this study population derived using the previously developed population PK model.

The previously developed population PK models of ipatasertib and its primary metabolite, M1, captured the parent and metabolite PK observations well in the IPATential150 study. In the previous population PK analysis [16], a typical value of AUC_SS_ for ipatasertib for a dose of 400 mg in the presence of abiraterone coadministration was estimated to be 3560 ng h/mL, with a 90% range of 1560–6734 ng h/mL, which indicated that the AUC_SS_ estimates for ipatasertib 400 mg QD oral dose in combination with abiraterone 1000 mg QD plus prednisone/prednisolone 5 mg BID obtained from the IPATential150 study population (Fig. 1) were consistent with the prior knowledge. This is also in line with the fact that the IPATential150 study population had similar distributions of baseline body weight and age, which were reported as influential covariates of PK of ipatasertib and M1; median (ranges) of baseline body weight and age in the active treatment arm of the IPATential150 study population were 81 (46–143) kg and 69 (47–93) years, whereas those in the population used for the population PK model development were 75 (41.5–160) kg and 64 (26–88) years.

The planned per protocol nominal dosing of ipatasertib 400 mg QD was used to derive exposure metrics using the previously developed population PK model in the primary exposure–response analyses in the present study. This was primarily to avoid potential post-randomization bias in the analyses; if the probability of a dose adjustment or interruption is correlated with exposure (i.e., higher exposure results in higher incidence of AEs and subsequent dose modification), the derived relationship between exposure and response will be biased relative to the true underlying relationship that would be obtained in the absence of any dose reductions. In fact, a significant number of patients experienced AEs leading to treatment discontinuation (*n* = 116, 21.1%), dose reduction (*n* = 220, 39.9%), or dose interruption (*n* = 319, 57.9%) in the active treatment arm of the IPATential150 study [15]. The results of the exposure–response analyses should be interpreted with caution due to this fact.

In general, exposure–efficacy and –safety results in the Phase 3 IPATential150 study were similar to those in the Phase 2 A.MARTIN study [13, 14], although it should also be noted that the patient populations in these two studies were different (first line vs second or later line settings). A statistically significant improvement in rPFS was not shown in the A.MARTIN study, though the study was designed for hypothesis generation and did not have adequate power to detect clinically meaningful differences, but the rPFS benefit was confirmed in the larger IPATential150 study which was adequately powered to address this question. All the safety endpoints of interest were associated with ipatasertib treatment or exposure.

Kaplan–Meier plot of rPFS in patients with PTEN-loss tumors stratified by placebo and quartiles of ipatasertib exposure indicated a beneficial effect of ipatasertib treatment (Fig. 2). However, the analysis was generally not indicative of any apparent exposure–response trends across the exposure quartile groups. The final multivariable Cox proportional hazards model suggested a significant correlation between ipatasertib AUC_SS_ and improved survival in patients with PTEN-loss tumors (HR: 0.92 per 1000 ng h/mL AUC_SS_, 95% CI 0.87–0.98, *p* = 0.011) (Fig. 3B). The confirmation of rPFS benefit in the Phase 3 IPATential150 study showcases the usefulness of conducting a well-designed randomized dose-ranging study (such as the Phase 2 A.MARTIN study), even if it is not statistically powered for efficacy demonstration, to properly assess the dose–exposure–response relationships and to inform the dose selection for the pivotal study in oncology drug development. In contrast, a significant improvement in subjects receiving ipatasertib (HR: 0.84, 95% CI 0.71–0.99, *p* = 0.038) was suggested in the ITT population, while ipatasertib exposure was not retained in the final model for this population.

There are some caveats to interpret this weak or lack of apparent exposure–efficacy trend of ipatasertib on rPFS; (i) as mentioned above, unscheduled dose adjustments occurred in a substantial number of patients by experiencing AEs leading to treatment discontinuation, dose reduction, or dose interruption in the active treatment arm of the IPATential150 study which makes exposure–response analysis challenging, and (ii) the fact that only one dose level was observed and studied in this pivotal study of ipatasertib. To address the first point, longitudinal analysis which accounts for the time-varying ipatasertib dose was performed. This approach was expected to allow better characterization of the true underlying exposure–efficacy relationship of ipatasertib. The results suggested that dose modifications might not have a large impact on treatment benefit of ipatasertib, to the extent and proportion of dose adjustments observed within the IPATential150 study.

Some of the other covariates retained in the final exposure–efficacy model also seem to be reasonable in terms of clinical relevance. Patients who had only PSA as a factor of progressive disease before initiation of the study treatment (i.e., progressive disease before initiating study treatment defined by two rising PSA levels measured ≥ 1 week apart and without radiographic evidence of disease progression) tend to show better rPFS outcome independently of treatment during the study period. Deterioration of overall health status, represented as ECOG status at baseline ≥ 1 and presence of visceral metastasis, was generally associated with reduced rPFS. Of note, tumor PTEN-loss was associated with worse rPFS when evaluated in the ITT population in the present study (data not shown) and was consistent with the previous reports [4–12].

Incidences of some AEs had statistically significant association with ipatasertib AUC_SS_ (SAEs, AEs leading to discontinuation, and Grade ≥ 2 hyperglycemia), while others were associated with only treatment effect and less sensitive to ipatasertib exposure (AEs leading to dose reduction, Grade ≥ 3 diarrhea, and Grade ≥ 2 rash) (Table 2). It seems reasonable that hyperglycemia especially showed an exposure–response relationship, because glucose homeostasis is a direct effect of the PI3K pathway and can be used as a pharmacological marker of AKT inhibition. Exposure–response relationships for diarrhea and rash, which had indicated a statistically significant association between ipatasertib exposure in the previous exposure-safety evaluations based on the Phase 2 A.MARTIN data [14], were less conclusive in the present study. This may be due to the fact that narrower dose/exposure ranges of ipatasertib was investigated in the Phase 3 IPATential150 study (which is the confirmatory trial investigating only one dose level of ipatasertib) compared with the Phase 2 A.MARTIN study (which is the dose-ranging trial investigating two dose levels of ipatasertib). Given that the planned per protocol nominal dosing of ipatasertib was assumed for deriving the exposure metrics in the present study, the results indicate that patients with characteristics that lead to higher ipatasertib exposure (e.g., lower volume of distribution due to lower body weight, decreased clearance due to greater age) would have higher probability to experience those AEs. To maintain the dose and maximize the efficacy of ipatasertib, early and optimal prophylactic measures to prevent ipatasertib dose reductions or discontinuations need due consideration.

In conclusion, this study characterized the exposure–efficacy and –safety relationships of ipatasertib in patients with mCRPC in the IPATential150 study. The exposure–efficacy results indicated that patients receiving ipatasertib may continue benefiting from this treatment at the administered dose, despite some variability in exposures. While ipatasertib was shown to be efficacious, results from the exposure–safety analysis suggested that the probability of AEs focused on in this study, such as hyperglycemia, diarrhea and rash, increases with ipatasertib treatment and/or increased ipatasertib exposures.

## Supplementary Information

Below is the link to the electronic supplementary material.

Supplementary file1 (DOCX 843 KB)

## Data Availability

Qualified researchers may request access to individual patient-level data through the clinical study data request platform: https://vivli.org/. Further details on Roche's criteria for eligible studies are available here: https://vivli.org/members/ourmembers. For further details on Roche’s Global Policy on the Sharing of Clinical Information and how to request access to related clinical study documents, see here: https://www.roche.com/research_and_development/who_we_are_how_we_work/clinical_trials/our_commitment_to_data_sharing.htm.
