# Supplementary material for: Characterization of exposure–response relationships of ipatasertib in patients with metastatic castration-resistant prostate cancer in the IPATential150 study
Source: Cancer Chemother Pharmacol. 2022 Oct 28;90(6):511–21. doi: 10.1007/s00280-022-04488-2 (PMC9637074; doi:10.1007/s00280-022-04488-2)

**Supplementary Materials for:**

**Characterization of exposure-response relationships of ipatasertib in patients with metastatic castration-resistant prostate cancer in the IPATential150 study**

(Cancer Chemotherapy and Pharmacology)

Naoki Kotani (1,2), Justin J. Wilkins (3), Janet R. Wade (3), Steve Dang (1), Dhruvitkumar S. Sutaria (1), Kenta Yoshida (1), Sameer Sundrani (1, 4), Hao Ding (1), Josep Garcia (5), Heather Hinton (5), Rucha Sane* (1), Pascal Chanu* (6)

* Co-senior authors of this manuscript

(1) Genentech, Inc., South San Francisco, CA, USA

(2) Chugai Pharmaceutical Co., Ltd., Tokyo, Japan

(3) Occams, Amstelveen, The Netherlands

(4) Department of Bioengineering / Biomedical Computation, Stanford University, Stanford, CA, USA

(5) F. Hoffmann-La Roche AG, Basel, Switzerland

(6) Department of Clinical Pharmacology, Genentech/Roche, Lyon, France

**Supplementary Figure S1**

Standard goodness-of-fit plots for the ipatasertib PK dataset from the IPATential150 study. Filled circles represent the individual patients. Solid hairlines connect each individual. Dashed orange lines are loess smooths. Black lines are lines of identity.


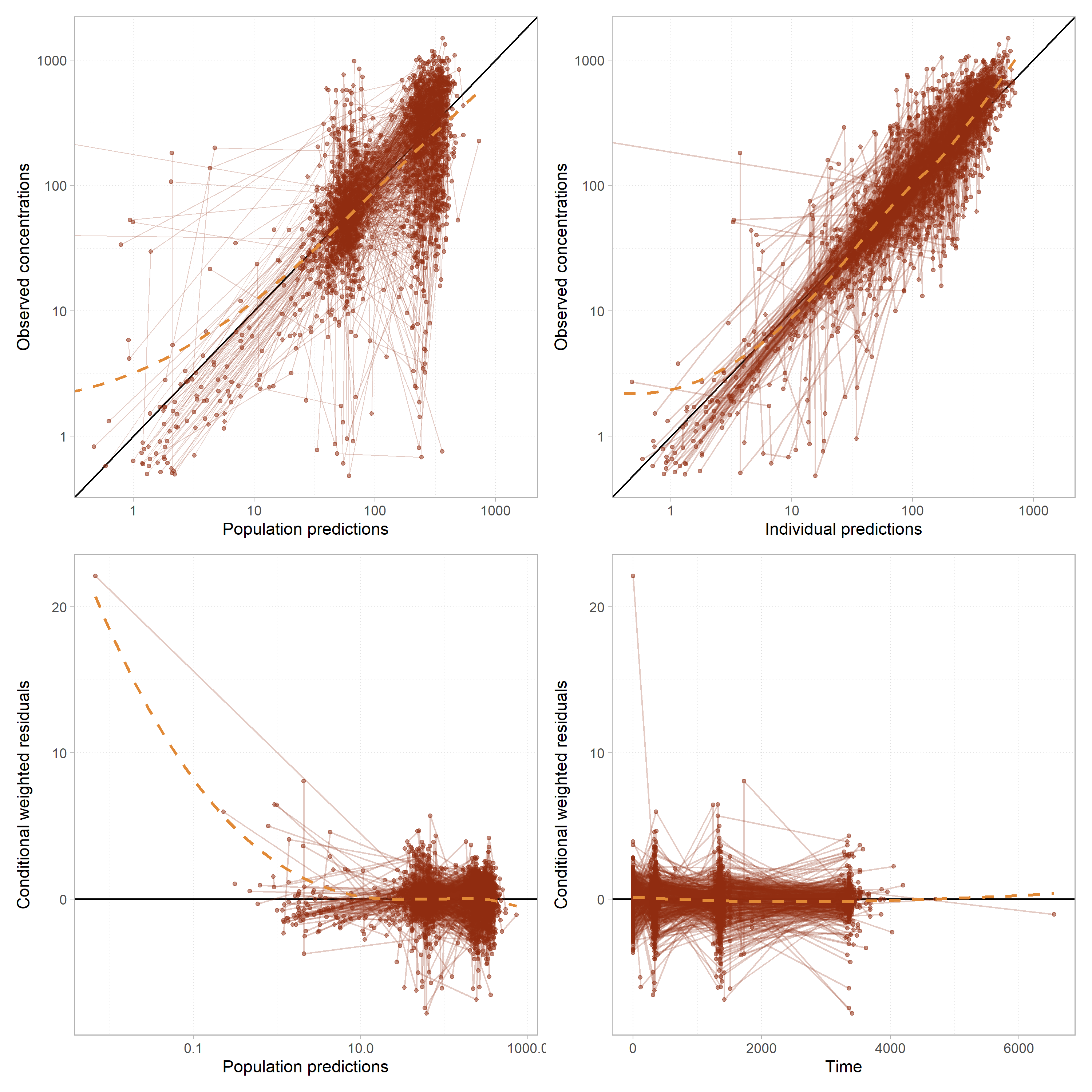


**Supplementary Figure S2**

Visual predictive check of PK data for the subjects in the IPATential150 study. Red filled circles represent the observed data. Orange and white points represent the observed medians (large circles), interquartile ranges (small circles), upper and lower limits of 90% range (small triangles). Shaded areas are violin plots of model predictions. Horizontal lines are predicted medians and 5th, 25th, 75th, and 95th quantiles.


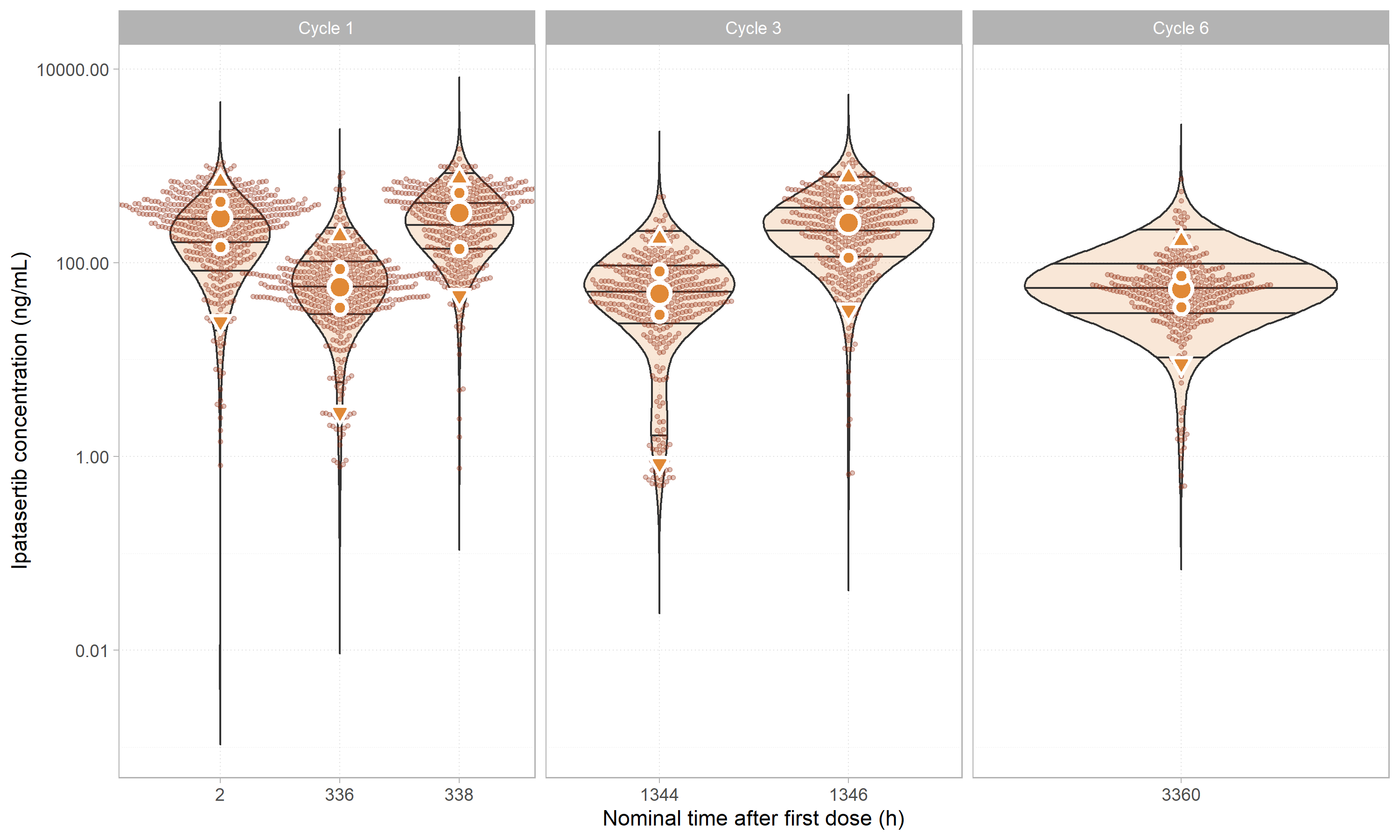


**Supplementary Figure S3**

Correlation of the simulated ipatasertib exposure metrics in the IPATential150 study population. AUC_sd_ = area under the concentration-time curve after single dose; AUC_ss_ = area under the concentration-time curve at steady-state; C_maxsd_ = maximum concentration after single dose; C_maxss_ = maximum concentration at steady-state; C_minsd_ = trough concentration after single dose; C_minss_ = trough concentration at steady-state.


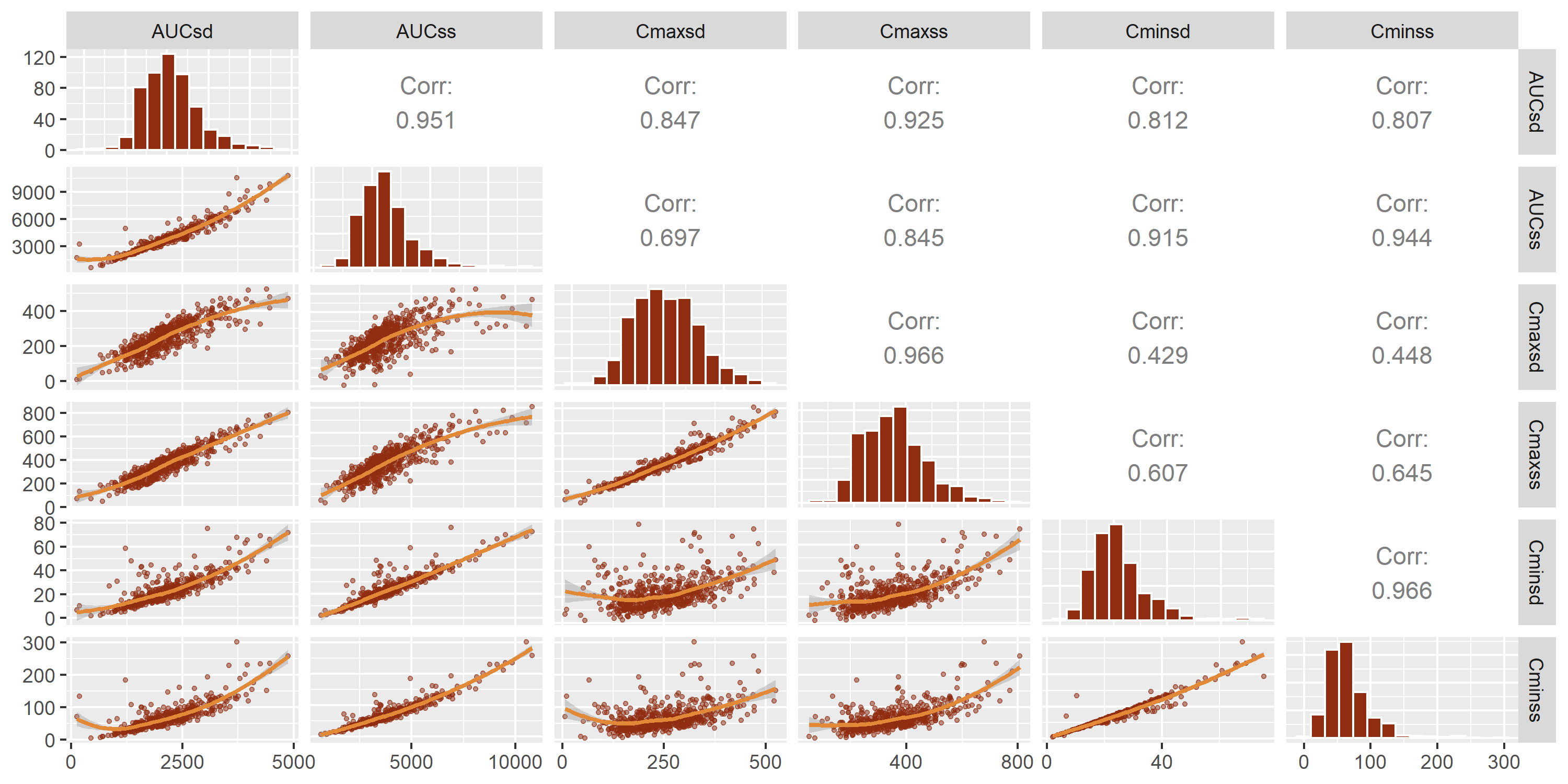


**Supplementary Figure S4**

AUC_SS_ correlation comparison between ipatasertib and M1 (G-037720) in the IPATential150 study. Filled circles represent the simulated exposure of individual patients. Orange solid line represent linear smooth. AUC_ss_ = area under the concentration-time curve at steady-state.


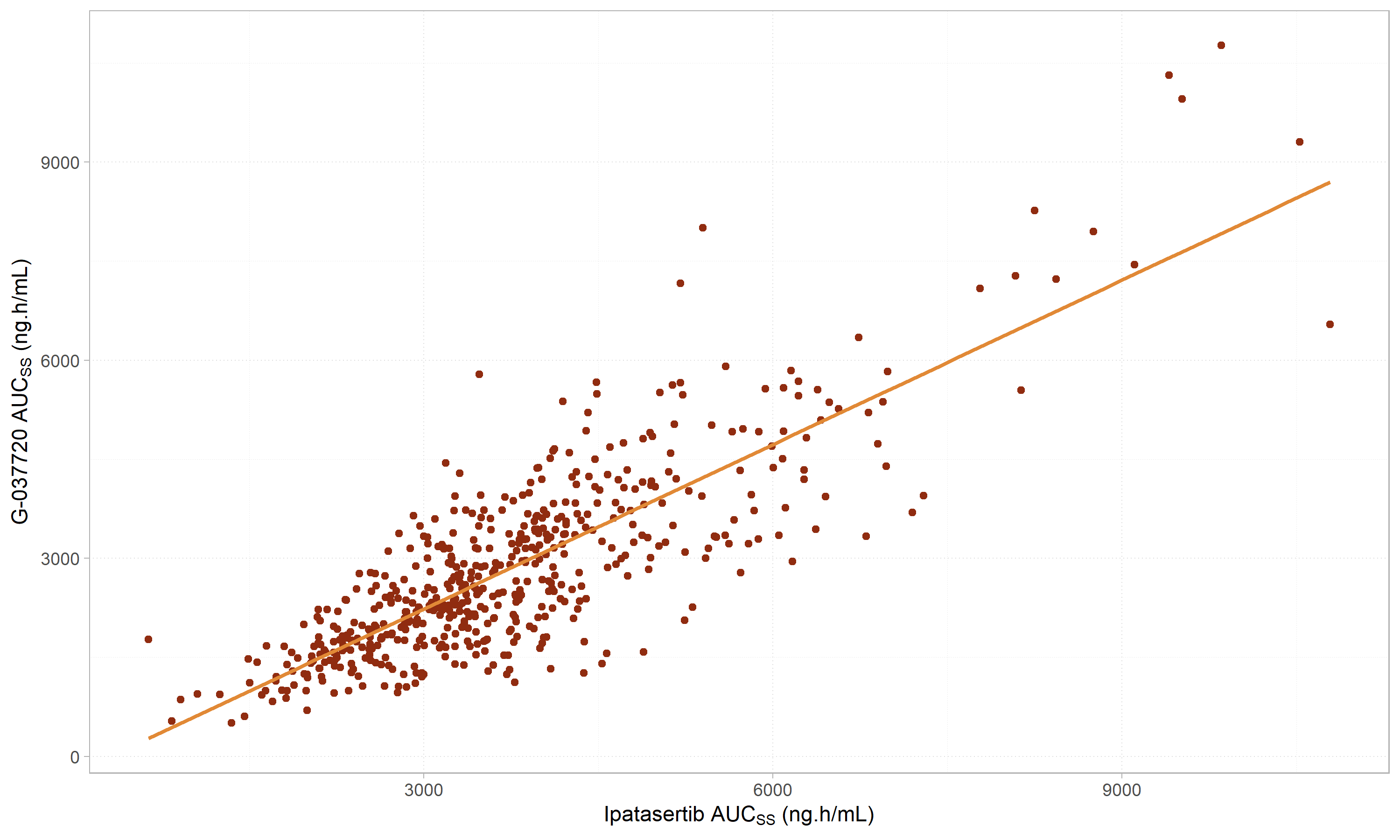

Supplement: Supplementary file 1 — Supplementary file1 (DOCX 843 KB) [file 280_2022_4488_MOESM1_ESM.docx]
